# Evaluation of Hepatoprotective Effect of Silymarin Among Under Treatment Tuberculosis Patients: A Randomized Clinical Trial

**Published:** 2016

**Authors:** Majid Marjani, Parvaneh Baghaei, Mehdi Kazempour Dizaji, Pegah Gorji Bayani, Fanak Fahimi, Payam Tabarsi, Ali Akbar Velayati

**Affiliations:** a *Clinical Tuberculosis and Epidemiology Research Center, National Research Institute of Tuberculosis and Lung Diseases (NRITLD), Shahid Beheshti University of Medical Sciences, Tehran, Iran.*; b *Mycobacteriology Research Center, National Research Institute of Tuberculosis and Lung Diseases (NRITLD), Shahid Beheshti University of Medical Sciences, Tehran, Iran.*; c *Chronic Respiratory Diseases Research Center, National Research Institute of Tuberculosis and Lung Diseases (NRITLD), Shahid Beheshti University of Medical Sciences, Tehran, Iran.*

**Keywords:** Tuberculosis, Adverse effects, Silymarin, Drug induced hepatitis

## Abstract

Abstract: Hepatic toxicity is the most serious adverse effect of anti-tuberculosis drugs. This study was performed to evaluate the efficacy of silymarin as a hepatoprotective herbal agent. In a randomized double blind clinical trial, 70 new cases of pulmonary tuberculosis were divided into two groups. The intervention group was assigned to receive silymarin and the control group received placebo. Tuberculosis was treated by classic regimen consisting isoniazid, rifampin, pyrazinamide and ethambutol. No statistically significant difference was found between the two groups concerning the frequency of drug induced liver injury or mild elevation of liver enzymes. Silymarin was safe without any major side effect. Our results showed no significant hepatoprotective effect of silymarin among patients on tuberculosis treatment.

## Introduction

Tuberculosis (TB) is a major concern of public health in the world with an estimated incidence of 8.6 million new cases in 2012 (1). Standard treatment of TB is a combination regimen consisting isoniazid, rifampin, pyrazinamid and ethambutol. The first three drugs are hepatotoxic and drug induced liver injury (DILI) is the most serious adverse effect among TB patients on treatment. Incidence of DILI has been reported between 2% and 28% (2, 3).

DILI may cause considerable morbidity or mortality (4). Furthermore, it may lead to discontinuation of the suspected agent(s) and significantly contributes to nonadherence, treatment failure, relapse, and drug resistance (2, 5-6). So, any preventive measure for decreasing risk of DILI is very attractive. 

Silymarin is a flavonolignan from *Silybum marianum*, a general medicinal herb of the Asteraceae family is being used from 4^th^ century B.C. It is a complex mixture of four flavonolignan isomers among them silybin is the most active component(7). Hepatoprotective activity of silymarin against some toxins and drugs has been showed by various animal studies and clinical trials (7-9). Although in rat models silymarin has been effective in prevention of adverse effects of anti TB drugs (10), to our knowledge there is not any published data about efficacy of the drug for that purpose in human.

We performed this study to evaluate potential hepatoprotective effect of silymarin among TB cases who received standard treatment.

## Experimental

*Setting and study population*

This study was performed at Masih Daneshvari hospital, the national referral center of tuberculosis and lung disease, Shahid Beheshti University of Medical Sciences, Tehran, Iran, from August to November 2010. In the period of study, newly diagnosed cases of tuberculosis, more than 18 years old, were recruited. Patients with concomitant HIV, HBV or HCV infection, preexisting liver disease, abnormal liver function tests (LFT) at the beginning of TB treatment, and pregnant and nursing mothers were excluded. 

*Study design and interventions*

The investigation was designed as double blind randomized clinical trial, which was registered in the Australian New Zealand Clinical Trial Registry, and the registry number is ACTRN12610000621011.

Treatment of tuberculosis was initiated by standard regimen consisting isoniazid (5 mg/kg), rifampin (10 mg/kg), pyrazinamide (20 mg/kg) and ethambutol (15 mg/kg).

After diagnosis of tuberculosis, the participants were randomly assigned with blocking to the treatment group or the placebo group by a researcher who was not directly involved in the trial. Group one was received Silymarin containing tablets (Livergol^®^) manufactured by Goldaru, Isfahan, Iran, three times per day for two weeks. Each Livergol^®^ 140 tablet contains dried extract of Sylibum marianum equivalent to 140 mg Silymarin. The second group was received placebo with the same shape, size and dose intervals manufactured by the same company. Drugs and placebo were encoded until analysis of results was done. 

*Ethical approval*

Ethical permission for the study was obtained from the ethics review board of the National Research Institute of Tuberculosis and Lung diseases. Written informed consent was obtained from all participants. 

*Primary and secondary o* *utcome* *measures*

Primary outcome was drug induced liver injury. Liver function was being evaluated at the beginning of treatment and three times per week for 2 weeks by measurement of serum aspartate aminotransferase (AST), alanine aminotransferase (ALT) and total bilirubin. Patients were examined and interviewed every day about any symptom or sign related to drug adverse effects (secondary outcome). After this period, liver function test (LFT) was evaluated if the patient had symptoms suspected to liver toxicity (below). Patients were followed up for six months.

Anti TB induced DILI was defined as 1) increasing of AST or ALT to three times more than upper limit of normal (40 IU/L), concomitant with symptoms of hepatic toxicity consisting nausea, vomiting, anorexia, weakness and abdominal pain 2) rise in AST or ALT more than five times or total serum bilirubin more than 2 mg/dl (2, 11). We considered any rise in liver enzymes less than the mentioned cut-off as “mild elevation of LFT”.

If DILI occurred, anti tuberculosis drugs and intervention (drug or placebo) were stopped and patients were managed using American thoracic society guideline (11).

The patients were strictly monitored for drug induced adverse effects including nausea, vomiting, diarrhea, vertigo, exanthema and other allergic phenomenon.

*Statistical analysis*

A chi-square statistic without Yates’ correction, Fisher’s exact test, the Student’s t-test and the Mann-Whitney U test were used as appropriate. Multivariate repeated measure analysis was used to adjust for potentially confounding factors affecting adverse outcome. All p-values were two-sided. A p-value less than 0.05 were considered to indicate statistical significance. Statistical analysis was performed using SPSS for windows (version 15.5).

**Figure 1:**
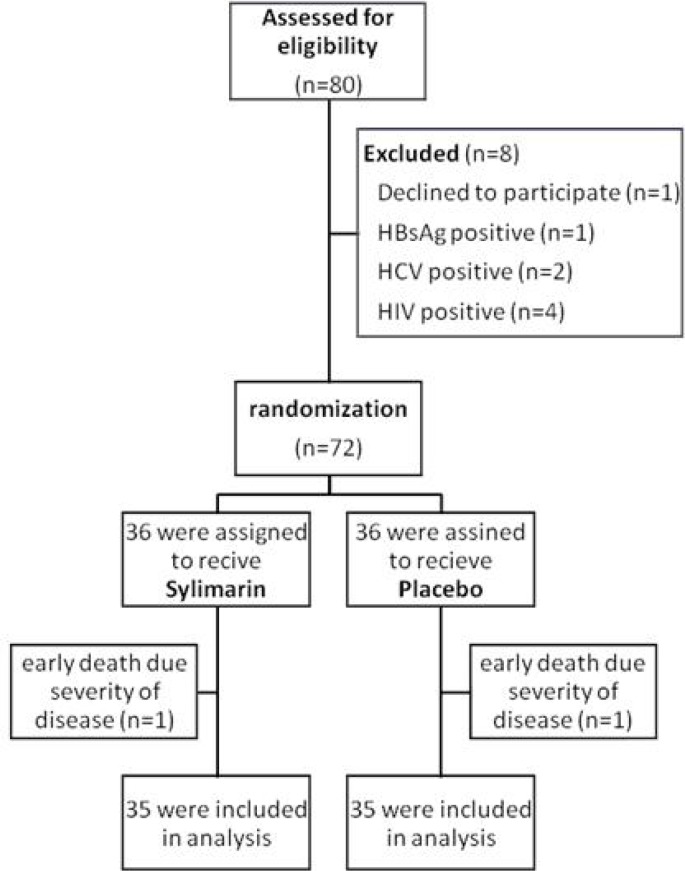
Enrollment, randomization, and follow-up of the study patients.

## Results

A total of 72 confirmed cases of tuberculosis underwent randomization. Patients were allocated equally either to the silymarin group or to the placebo group. One case of each group died after four and five days from respiratory failure related to severity of pulmonary tuberculosis. Finally, 70 cases were included in the analysis. (Figure 1) Among them, 37 (53%) were men and 33 were women with mean age of 49.86 ± 22.75 years. All of cases had pulmonary tuberculosis. There was not any statistical difference between two groups concerning sex, age, nationality, smoking status, opium addiction and concomitant diabetes mellitus. Patient’s characteristics were summarized in Table 1. 

The primary outcome was occurrence of anti TB drugs induced liver injury. Six patients of silymarin group (17.1%) and three of placebo group (8.6%) experienced DILI. It was not statistically significant (p-value= 0.239). One case of silymarin group and two cases of placebo group had mild elevation in the liver enzymes resolved spontaneously without any change in the regimen. Frequency of this phenomenon was not statistically different between the two groups (p-value=0.500). Also the difference between frequencies of gastrointestinal complaints was insignificant. In silymarin group 3 patients (8.8%) experienced pruritus and one case experienced mild maculopapular rash while the number if patients with pruritus were 5 cases (14.3%) in the placebo group. All of these complications were controlled by low dose hydroxyzine (10 mg three times per day) easily and no drug was necessary to discontinue. No statistically difference was found between the two groups concerning adverse drug effects. 

These findings are summarized in Table 2.

**Table 1:** Basic characteristics of two groups

	**Silymarin group**	**Placebo group**	p-value
Age			
mean <65 ≥65	50.1 23 (65.7%) 12 (34.3%)	49.6 23 (65.7%) 12 (34.3%)	NS
Nationality			
Iranian non Iranian	28 (80%) 7 (20%)	27 (77.1%) 8 (22.9%)	NS
Sex			
male female	19 (54.3%) 16 (45.7%)	18 (51.4%) 17 (48.6%)	NS
Smoking	7 (20%)	7 (20%)	NS
Opium addiction	7 (20%)	6 (17.1%)	NS
DM	3 (8.6%)	1 (2.9%)	NS
Total	35	35	

Abbreviations: DM: diabetes mellitus, NS: no significant

**Table 2:** Comparison of liver injury and side effects of drugs between two groups

	**Silymarin group**	**Placebo group**	p-value
Anorexia	4 (11.4%)	0	0.054
Nausea	2 (5.7%)	3 (8.6%)	NS
Vomiting	0	3 (8.6%)	NS
Abdominal pain	4 (11.4%)	3 (8.6%)	NS
Mild elevation of LFT	1 (2.9%)	2 (5.7%)	NS
DILI	6 (17.1 %)	3 (8.6%)	NS
Pruritus	3 (8.6%)	5(14.3%)	NS
Exanthema	1 (2.9%)	0	NS
Diarrhea	0	0	-
Vertigo	0	0	-
Total	35	35	

Abbreviations: LFT: liver function tests, DILI: drug induced liver injury, NS: no significant

## Discussion

In this analysis involving 70 new cases of tuberculosis, silymarin didn’t have any effect on decreasing the risk of drug induced hepatitis. Also frequency of mild elevation of liver enzymes was not different between patients who received silymarin and who didn’t receive it. The drug was safe, without any major side effect. 

Hepatoprotective effect of silymarin has been investigated by previous studies. It has been effective in prevention of hepatic toxicity caused by acetaminophen (12), carbon tetrachloride (8), ethanol (9) and amanita phalloides toxin (13). Most of these results found in animal models although few studies were performed in human showed efficacy of silymarin (7).

In a rat model by Eminzade and colleagues co-administration of silymarin together with isoniazid, rifampin and pyrazinamide significantly decreased hepatic toxicity of anti TB drugs. This effect revealed by assessment of histopathological changes caused by anti TB drugs and biochemical tests such as the levels of AST, ALT, alkaline phosphatase and bilirubin concentration. However silymarin could not efficiently protect against DILI (10).

To our knowledge, there is no previous published human study concerning effect of silymarin in prevention of DILI among under treatment TB patients. 

The exact mechanism of liver injury correlated to anti TB drugs is unknown. Toxic metabolites (like hydrazine for isoniazid) (14), oxidative stress (15) and hepatocellular dysfunction (16) are considered. Also limited data were found about genetic susceptibility (17-18). On the other hand antioxidant properties, stimulation of protein synthesis and anti-inflammatory effect proposed as the mechanisms that silymarin prevent hepatocellular damage (7, 19). The mechanism responsible for DILI related to anti TB drugs may be different from mechanism of hepatoprotection by silymarin. 

Good points of our study were usage of placebo and double blindness, although it had a limitation. It was performed in a referral center and results in the field may be different. 

TB patients with comorbidities such as HIV, HBV and HCV infections or preexisting liver diseases are more susceptible to DILI (20-22). We exclude these patients. So we cannot generalize the result of this study to the high risk population. 

## Conclusion

This study showed that silymarin does not have significant effect in prevention of hepatic toxicity of anti-tuberculosis drugs. Although silymarin is safe, routine usage of this herbal compound together with TB treatment is not justified. Additional studies are necessary to evaluate hepatoprotective effect of silymarin among special high risk groups. 
